# Prediction of Cable Behavior Using Finite Element Analysis Results for Flexible Cables

**DOI:** 10.3390/s23125707

**Published:** 2023-06-19

**Authors:** Hyeonjin Kim, Jinhyun Kim

**Affiliations:** Department of Mechanical Engineering, Seoul National University of Science and Technology, Seoul 01811, Republic of Korea; hyeonjin@seoultech.ac.kr

**Keywords:** finite element method, flexible cable, cable winding, material property

## Abstract

In actual industrial sites, verifying the framework for cable manipulation is crucial. Therefore, it is necessary to simulate the deformation of the cable to predict its behavior accurately. By simulating the behavior in advance, it is possible to reduce the time and cost required for work. Although finite element analysis is used in various fields, the results may differ from the actual behavior depending on the method of defining the analysis model and analysis conditions. This paper aims to select appropriate indicators that can effectively cope with finite element analysis and experiments during cable winding work. We perform finite element analysis of the behavior of flexible cables and compare the analysis results with results from experiments. Despite some differences between the experimental and analysis outcomes, an indicator was developed through trial and error to align the two cases. Errors occurred during the experiments depending on the analysis and experimental conditions. To address this, weights were derived through optimization to update the cable analysis results. Additionally, deep learning was utilized to update the errors caused by material properties using the weights. This allowed for finite element analysis even when the exact physical properties of the material were unknown, ultimately improving the analysis performance.

## 1. Introduction

The field of automated robots that can replace humans in performing repetitive tasks in various industrial fields is rapidly growing. By using robot manipulators, dangerous and difficult tasks can be performed more quickly and precisely. Work performance can also be improved by optimizing work paths through simulation. To accurately move the manipulator in the simulation workspace, it is crucial to understand the movement of the end effector, on which the work tool is mounted, and the characteristics and behavior of the object being worked on.

Problems related to the deformation of flexible objects frequently occur in industrial sites, and in particular, work using flexible cables, such as cable arrangement and pick-and-place, is frequently performed. Although humans can easily manipulate cable deformation, the increasing use of robot manipulators has made it important to verify the framework for cable manipulation to replace humans with robots [1].

In cable winding work, which is a typical example of using manipulators in the manufacturing process, the work path changes depending on cable deformation. Inaccurate simulation of the movement during manipulation can lead to errors in the manipulator’s work, resulting in reduced production efficiency. Therefore, it is necessary to accurately represent the behavior of flexible cables during simulation to match actual behavior. However, due to the nonlinear changes in physical properties, accurately understanding the properties of flexible cables has its limitations. Neo-Hookean and Mooney–Rivlin models are used for typical nonlinear analysis such as cable analysis [2,3]. In addition, nonlinear material behavior can be expressed as a stress–strain relationship using other coefficients [4]. However, nonlinear elastic models have complex parameters to consider, which can affect the accuracy of the model. Therefore, if sufficient experience and knowledge are lacking, large errors may occur in the analysis result or there is no convergence. On the other hand, this model uses only elements such as Young’s modulus and Poisson’s ratio and can accurately model the material’s elastic behavior within a small deformation range.

In product design and experimentation, the finite element method can predict results in advance, helping to save time and costs [5]. The finite element method is a numerical analysis technique used to solve continuum problems and has been widely utilized in various fields such as mathematics, physics, and engineering [6,7,8]. However, when there is insufficient theory or data required for interpretation, the practical use of this method in solving real engineering problems becomes challenging. For example, calculating the complex stress state that continuously changes in fatigue failure lacks sufficient theory, while the lack of data on the thermal and mechanical properties of non-metallic materials limits finite element analysis for non-metallic materials [9,10].

Although finite element analysis using software is common in various fields, the reliability of the results obtained may vary depending on the method used to define the analysis conditions, assuming a model similar to the real world [11]. In order to enhance the performance of analytical models, it is essential to verify the finite element-based model with sufficient experimental data and improve the correlation between the simulation and experiment [12].

Several methods to verify the accuracy of finite element analysis results involve using sensors or vision systems [13,14]. For example, sensors can be placed on the cable to measure its deformation or other physical properties during an experiment. Similarly, vision systems can capture images of the cable and provide accurate measurements of its deformation. By comparing the results of the experiment with the results of the finite element analysis, the accuracy of the analysis can be assessed. It is important to note that different verification methods may be appropriate for different types of cables or materials. For example, for flexible cables, vision systems may provide a more accurate measurement of deformation than sensors. Therefore, it may be necessary to use a combination of verification methods to obtain the most accurate results.

Another important consideration when verifying finite element analysis results is the development of appropriate verification indices. These indices can be used to evaluate the accuracy of the analysis and identify areas where improvements are needed. Overall, experimental verification is an important step in the finite element analysis process. Especially for flexible cables and other materials with complex behavior. By carefully selecting appropriate verification methods and indices, the accuracy of the analysis can be improved, and the reliability of the results can be ensured.

In this study, a method for experimentally verifying the finite element analysis model for flexible cables is discussed. The input and output variables of the analysis and experimental model were set, and an index was developed to verify the finite element analysis results. The experimental environment was set to be the same as the finite element analysis, and verification methods based on sensors and vision systems were used. Vision systems are particularly effective in observing deformation of objects, making them useful for comparing flexible cable analysis and experimentation results. This approach has the potential to improve productivity and economic feasibility by predicting various problems at industrial sites through simulation. Therefore, this paper aims to improve the efficiency of automation work with flexible bodies by predicting the behavior of flexible cables close to reality using only finite element analysis results and applying optimization using machine learning. In this paper, a relatively simple linear elastic model is employed to obtain simulation results. Through comparison with experiments, the errors resulting from nonlinear material properties are identified, and the proposed approach suggests using machine learning techniques to correct for these errors.

## 2. Materials and Methods

Automating the process of winding flexible cables around a cylinder can be challenging due to the varying behavior of the cables depending on their material properties. Finite element analysis can be utilized to understand the cable behavior for a specific material, and the results can be used as input data for machine learning algorithms to predict the behavior of cables made from different materials. To ensure the accuracy of the finite element analysis results, indicators can be set up and experiments can be conducted to validate the analysis. This approach can help predict the behavior of flexible cables for various materials, enabling the automation of cable winding processes. In this study, for the analysis and experimentation with the flexible cable, a material exhibiting axial length changes was utilized to account for the cable’s axial rigidity. The analysis and experiments were performed using a flexible cable made of silicone rubber. The density ($\rho =1.337\times 10^{-9}[\frac{tonnes}{mm^{3}}]$) was calculated by measuring the volume and mass of the actual silicone rubber cable. Poisson’s ratio (ν=0.47) and elastic modulus (E=2.6 MPa) were measured through a tensile test.

### 2.1. Configuration of Model Parameters for Verification of Finite Element Analysis Results

**Figure 1:**
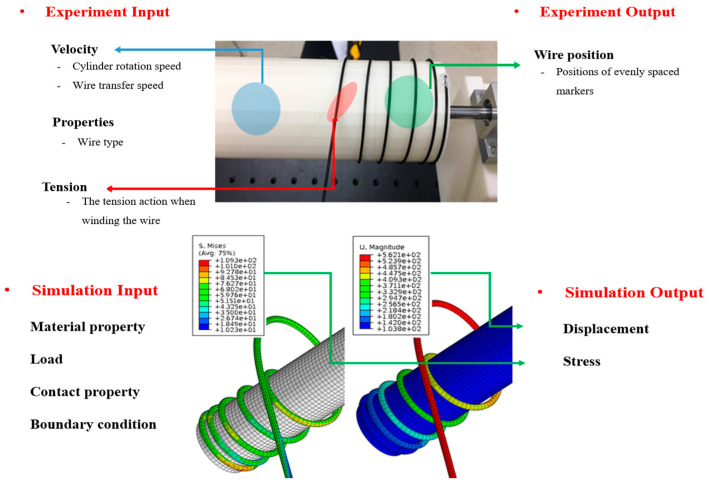
Parameters of experiment and finite element analysis for flexible cable.

In order to ensure the accuracy and reliability of the finite element analysis results for flexible cables, it is essential to validate them through experiments. To achieve this, it is crucial to establish a correlation between the results of the finite element analysis and those of the experiments. In other words, it is necessary to select an appropriate analysis model that can effectively capture the behavior of the cable under different conditions and accurately predict its response. For flexible cable operations, as shown in Figure 1, the output value is determined based on the input variables of the finite element analysis, and the input and output variables in the experiment are also checked. These variables can include factors such as the material properties of the cable, the winding angle, and the applied tension. Once the input and output variables have been identified, the parameters of the analysis model are configured by combining the input and output variables that can be commonly utilized in both the finite element analysis and experiments. By selecting appropriate input and output variables and establishing an effective correlation between the finite element analysis and experimental results, it is possible to validate the accuracy of the analysis model and further improve its reliability.

#### 2.1.1. Model for Finite Element Analysis

**Figure 2:**
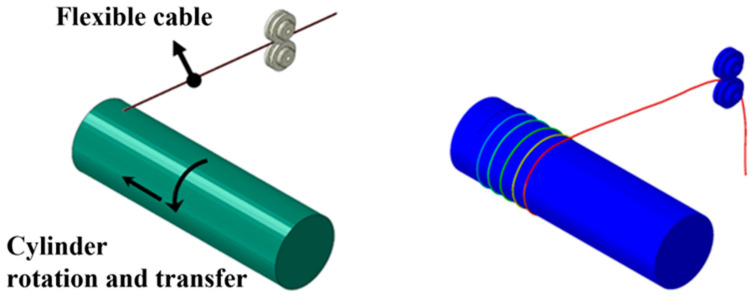
Finite element analysis model of flexible cable.

During the preprocessing stage of finite element analysis, the problem is defined and the analysis model is formulated to incorporate the analysis conditions such as the material properties, contact conditions, initial conditions, and boundary conditions. The domain variable values are then calculated by combining the governing equations in the form of a stiffness matrix. Based on this, the induction variables are calculated to obtain a solution, such as stress distribution, deformation shape, and reaction force. To verify the accuracy of the finite element analysis model, the shape change of the cable, which is a common result of the finite element analysis and the experiment of winding a flexible cable around a cylinder, was compared. With the end of the flexible cable fixed to the cylinder, the cylinder rotates and is transported in one direction, and the cable is wound around the cylinder. Even when cable winding work is performed in actual industrial sites, the manipulator only serves to adjust the initial position of the cable and cut the cable, and the cylindrical shape rotates to wind the cable, which is automated. Therefore, to perform finite element analysis under the same conditions as the experiment, an analysis model was created in the form shown in Figure 2 with the flexible cable end fixed to the cylinder, which rotates and is transported in one direction while the cable is wound around it.

#### 2.1.2. Conditions of Finite Element Analysis

**Figure 3:**
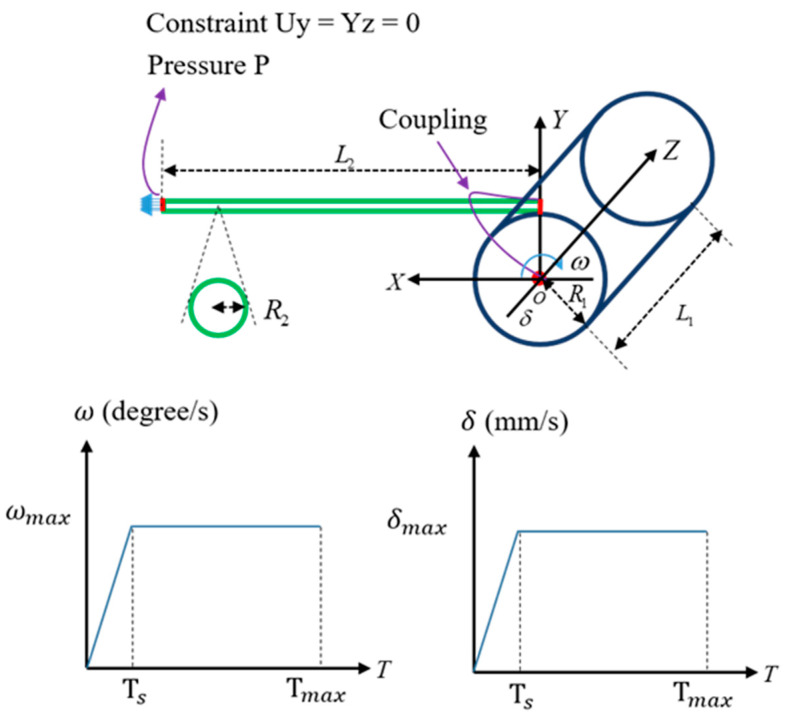
ABAQUS model of cable winding.

In order to simulate the winding of the flexible cable around the cylinder, a finite element analysis of the flexible cable was performed using ABAQUS, as shown in Figure 3. Since the change in the shape of the cable according to the material properties of the flexible cable is important, the flexible cable was analyzed by dividing the elements into an 8-node Reduced Integration Element (C3D8R) and setting the cylinder as an Analytic Rigid Surface. To improve the speed of finite element analysis, the element sizes were adjusted based on the cable diameter. In the radial direction, 12 nodes were positioned along the outer circumference of each cable cross-section. In the axial direction, the node spacing was set to approximately five times the cable diameter. For cables with a diameter of 2 mm, a node spacing of 10 mm was used in the axial direction. When the cable diameter increased to 4 mm, the axial node spacing was adjusted to 20 mm. In the case of a cable diameter of 6 mm, an initial axial node spacing of 30 mm was employed, following the same ratio as the previous cases. However, this configuration resulted in unacceptably slow analysis speed. As a result, the axial node spacing was modified to 50 mm to achieve a reasonable compromise between analysis speed and accuracy. By tailoring the element sizes according to the cable diameter, the finite element analysis could be performed more efficiently without compromising the essential features and behavior of the cables. The contact condition between the cylinder and the cable was general contact, and the dynamic/explicit analysis method was applied with the friction coefficient set to 0.1. The explicit finite element analysis method is an inertial force-based analysis method. It is suitable for problems with strong nonlinearity because it obtains a solution by substituting the value of the previous step into the next step without solving the stiffness matrix. In order to obtain a stable solution, the time interval is shortened to derive output variables such as acceleration, velocity, and reaction force over time, so it has the advantage of high convergence. The cylinder was set to rotate at a speed of $\omega_{max}=1.74\;rad/s$, while the feed speed was $\delta_{max}=5\;mm/s$. The speed increased linearly until 1 s, and the analysis was conducted at the same speed for 20 s after 1 s. $T_{s}$ represents the time it takes to reach the maximum speed, while $T_{max}$ represents the time it takes for the cable rotation and translation to be completed.

(1) $$\tan\lambda =\frac{l}{\pi D}=\frac{l}{2\pi R}$$

(2) l=2πR×tanλ

(3) $$\Delta S=\sqrt{2\pi {\left(R+r\right)}^{2}+l^{2}}$$

#### 2.1.3. Metrics for Verifying Finite Element Analysis Results

**Figure 4:**
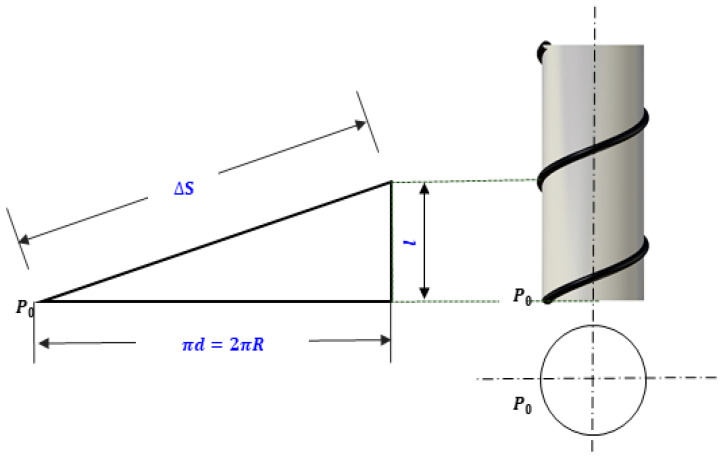
Calculation methodology for determining the marker spacing to be displayed on the cables.

To validate the accuracy of finite element analysis for flexible cables, an appropriate index for comparing analysis results with experiments is required, as shown in Figure 4. The marker spacing (∆S) was determined by using the cylinder radius (d), cable radius (r) and the pitch of the cable (l) when it was wound around the cylinder, with the starting point ($P_{0}$) of the cable fixed on the cylinder as the reference. This marker spacing was then set as an indicator to determine the length of the cable. When performing finite element analysis using input variables such as material properties, initial tension, and winding speed of the flexible cable, the shape of the cable is subject to change. During finite element analysis, it is easy to obtain the displacement after cable deformation since the coordinates of all nodes before and after analysis can be determined. However, there are limitations to measuring the 3-dimensional displacement in experiments. To overcome this, markers were attached to the flexible cable, and the displacement of the cable was measured based on the coordinates of the center of the marker to capture the shape change. By marking the cable at regular intervals and performing the experiment, the center coordinates of the markers could be obtained by recognizing the markers using OpenCV. To determine the displacement of the flexible cable in the experiment, the coordinates were obtained by calculating the cable length using the distance between the markers and the diameter of the cylinder and flexible cable.

To collect the coordinate values (x,y,z) of the corresponding node from the analysis result after cable deformation, the node number of the element located at the same position as the marker before deformation was first identified. The accuracy of the experiment could be verified by converting the collected Cartesian coordinate data into a cylindrical coordinate system and comparing it with the analysis results.

The position of each marker was determined based on the diameter of the flexible cable, the diameter of the cylinder, and the distance between the cables when the cable was wound. Each marker was positioned within the same frame when the cable was wound around the cylinder. The centers of all markers are theoretically located at the intersection of the tangent plane containing the starting points of the cylinder and the cable.

### 2.2. Configuration of Experiment Environment

During the experiments, a manipulator can be used to control the path of the cable, allowing it to wind along a specific trajectory. However, when using the displacement of the cable as an input variable in finite element analysis, the output variable becomes a stress value that cannot be directly compared with experimental measurements. Additionally, maintaining a constant tension during cable winding is difficult to achieve in experimental environments, while it is possible to simulate it using finite element simulations. 

**Figure 5:**
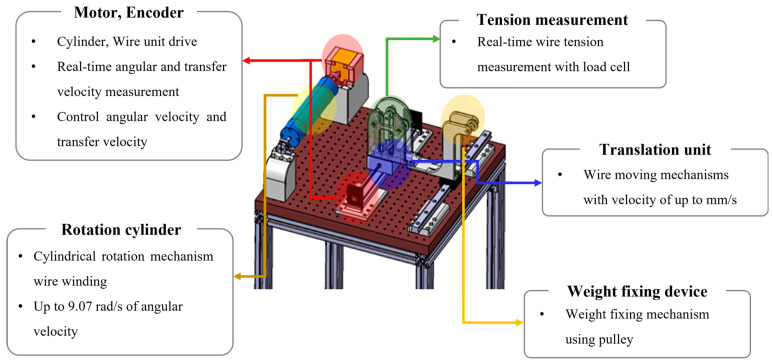
Schematic diagram of the experiment.

To validate the simulation results experimentally, an experimental setup was designed as shown in Figure 5. A linear motion device was used to transport the flexible cable in the axial direction of the cylinder while the cylinder rotated at a constant speed. This method allowed the cable to be wound around the cylinder, creating the same experimental environment as the simulation. Using this approach, simulation and experimental results could be more accurately compared in this study.

#### 2.2.1. Design of Experimental Equipment

**Figure 6:**
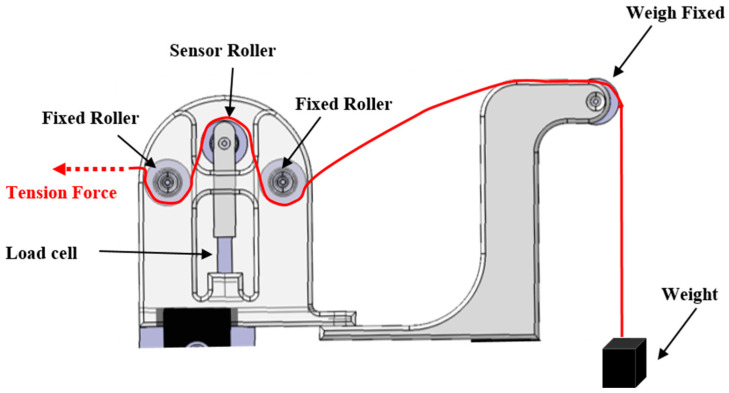
Module configuration for tension measurement.

**Figure 7:**
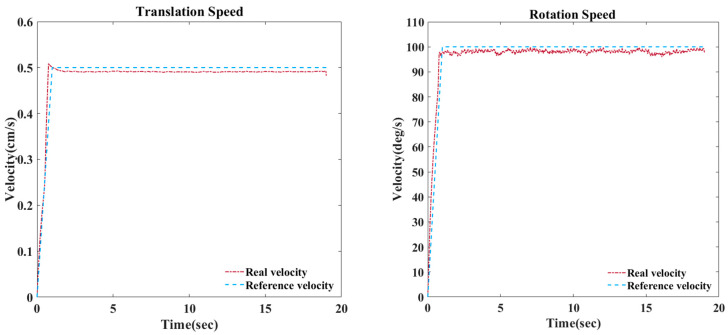
Graph of translation and rotation speeds used in cable winding experiments.

A mechanism for rotating the cylinder and a mechanism for moving the position of the wire were produced and constructed, and each mechanism was equipped with an encoder and a motor to enable real-time control. The system that controlled the rotation and transfer speed was configured to maintain a constant speed by transmitting the measured value from the speed measurement MCU to the motor control MCU. The MCU for speed measurement operated at 1000 Hz, and the MCU for motor control operated at 500 Hz, so it was possible to operate quickly and accurately. Figure 6 shows that, to maintain a constant tension while winding the flexible cable around the cylinder, a weight was attached to the end of the cable, and a load cell was attached to the cable transfer device to measure the initial tension and tension during the experiment [15,16]. In addition, the design allowed the cable to move smoothly using pulleys to minimize friction while the cable was wound around the cylinder. In the experiment, the rotational speed of the cylinder and the conveying speed of the wire were controlled through the PID algorithm [17,18] to ensure they were identical to the conditions of the finite element analysis. As shown in Figure 7, it was confirmed that the target value was closely followed. Reliability was improved by matching the conditions of the analysis and experiment.

#### 2.2.2. Cable Displacement Measurement Using Vision System

**Figure 8:**
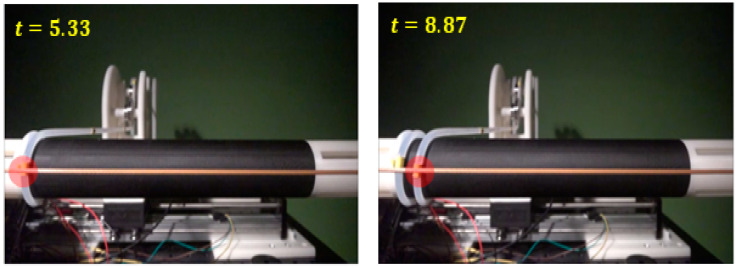
Image of the experimental process.

After fixing one end of the flexible cable to the cylinder, it was transferred in the axial direction of the cylinder and the displacement of the cable was measured using a high-speed camera while rotating the cylinder, as shown in Figure 8. During the cable winding, the coordinates (r,θ,z) of the marker center point were detected using the cylinder axis transfer distance and the diameter of the cylinder and cable. The high-speed camera used in the experiment can measure 1000 FPS at a resolution of up to 1920 × 1080 (FHD) and 24,046 FPS at a resolution of 640 × 96. The marker center coordinates were calculated by calculating the rotation speed of the cylinder and the number of frames of the high-speed camera. At this time, the distance from the center of the actual camera to the object changes due to the distortion of the camera lens, so the exact distance was corrected [19,20]. To accurately detect the position of the yellow marker attached to the cable, we used a specific color algorithm in OpenCV to detect its color [21,22,23]. We converted the marker’s RGB value to an HSV value to configure the algorithm for color classification. To prevent any potential issues with detecting objects with colors similar to the marker in the experimental environment, we extracted the RGB values of the markers in the image to detect accurate marker color information and adjust the threshold accordingly.

#### 2.2.3. Selection of Flexible Cable Materials and Test Conditions

In the process of winding the flexible cable around the cylinder, the axial rigidity of the cable was taken into account by using silicon rubber, which changes in length in the axial direction. For the actual experiment, the physical properties of the materials used were directly measured and used as input conditions for finite element analysis and experiment. The density was calculated by measuring the volume and weight of the cable, while the elastic modulus and Poisson’s ratio were measured using a tensile tester. Table 1 shows the experiment and finite element analysis conditions, which were varied by changing the cylinder diameter to 30 mm and 60 mm with cable diameters of 2 mm, 4 mm, and 6 mm. The experiment was also conducted with variations in the tension for the same cylinder diameter and cable diameter. 

## 3. Finite Element Analysis and Collection of Experimental Datasets

**Figure 9:**
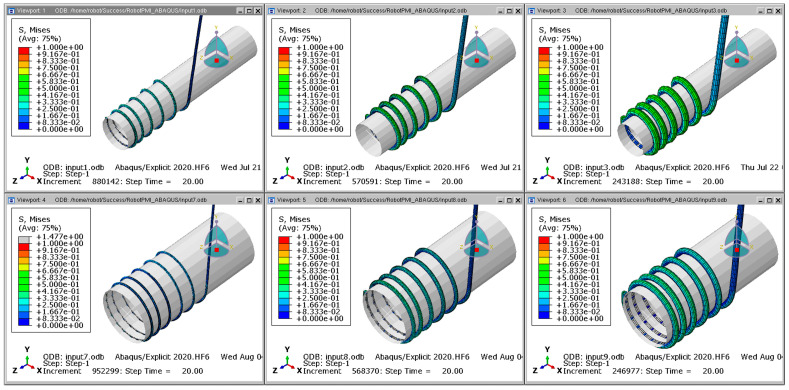
Results of finite element analysis for silicone rubber cable.

Ten experiment repetitions were conducted to verify the accuracy of the finite element analysis for a flexible cable under the same conditions. To enable comparison with the experimental results, the finite element analysis results in orthogonal coordinate systems were converted into cylindrical coordinate systems, and the curve length between the center coordinates of the fixed marker and each marker was calculated. The results of the finite element analysis were validated by comparing the curve length between marker center coordinates after cable deformation to the marker interval before deformation. In the experiment, the curve distance was calculated on a cylindrical coordinate system with the sum of the cylinder and cable radii as the radius because the cable was twisted while being wound around the cylinder. In the finite element analysis, the location of each node was compared while the cable was deformed by grasping the node corresponding to the same point as the marker’s position before the flexible cable was deformed. Figure 9 shows the results of the finite element analysis, which indicate that the axial length change decreased as the diameter of the flexible cable increased. Through this, it was possible to confirm the difference in axial stiffness through the axial length change that varies depending on the cable diameter.

### 3.1. Displacement Difference of the Center Marker Coordinates on the Flexible Cable

Through the experiments and finite element analysis, the angle differences and z-coordinate changes were measured to compare the behavior of the cable according to its diameter. The curve distance from the center coordinate of the fixed marker to the center coordinate of each marker was calculated using this information and the radius of the cylindrical coordinate system. The simulation results were verified through experiments. Table 2, Table 3 and Table 4 present the comparisons between the finite element analysis and experimental results while varying the diameter of the cylinder and the flexible cable. Table 2 shows the results for a cable diameter of 2 mm and Table 3 presents the results for a cable diameter of 4 mm. Similarly, Table 4 reports the results for a cable diameter of 6 mm.

### 3.2. Curved Length between Marker Center Coordinates Marked on a Flexible Cable

**Figure 10:**
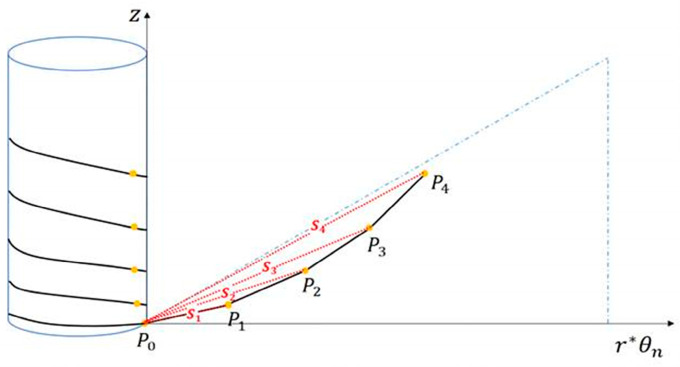
Curve distance between marker center coordinates.

Accurately calculating the length of the cable curve involved measuring the difference in angle and z-coordinate change, while also taking into consideration the diameters of both the cylinder and the cable. It is worth noting that torsion occurs when the flexible cable is wound around the cylinder, which can have an impact on the curve distance calculation. To overcome this challenge, a cylindrical coordinate system was utilized, where the radius was determined by adding the cylinder radius to the cable diameter. The calculated curve length was then compared with the length before the cable deformation, and this comparison was carried out through both finite element analysis and experimentation. The detailed methodology for measuring the curve length is illustrated in Figure 10, while the comparison of results is presented in Table 5 and Table 6.

### 3.3. Calculation of Length Error of Flexible Cable

Table 7 presents the error calculations obtained by comparing the curve length between the marker center coordinates in the finite element analysis and those of the experiment with a flexible cable, using the length before cable deformation. The accuracy of the finite element analysis was confirmed by calculating the relative error for each condition. The results indicate that the analysis and experimental outcomes are most consistent when the cable diameter is 4 mm. However, for a cable diameter of 2 mm, the cable length changes more in the experiment than in the analysis, while for a cable diameter of 6 mm, the length change is greater in the analysis than in the experiment.

## 4. Automatic Updating of Physical Properties of Flexible Cable through Machine Learning

The analysis models were categorized based on experimental and finite element analysis conditions such as the cylinder diameter, flexible cable diameter, and tension. To create a dataset for machine learning, the curve length from the fixed point to the marker was calculated by combining the flexible cable displacement obtained from finite element analysis with the coordinate changes of the markers positioned at regular intervals in the actual experiment. Each dataset was matched with a learning model by applying the K-NN (K-Nearest Neighbor) algorithm [24,25].

**Figure 11:**
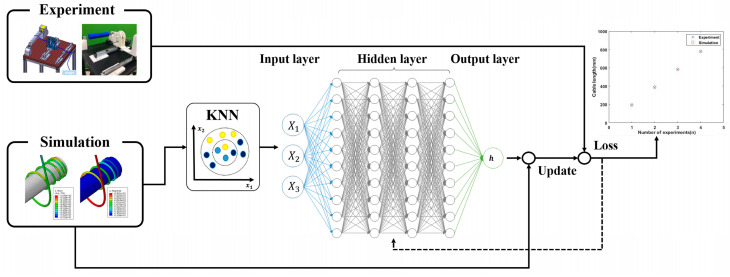
Network architecture for updating the cable length error.

The K-NN algorithm was used to analyze the cable curve length error data obtained from the experiments and finite element analysis, resulting in accurate classification of the experimental conditions based on the given cable length. The achieved classification accuracy was up to 91.3%. Using accumulated data classification and error automatic update with DNN, weights were obtained for new input data not included in the classified data. The input data included the cylinder diameter, cable diameter, tension, and others. The neural network was constructed with three hidden layers, each consisting of ten nodes, and ReLU (Rectified Linear Unit) was used as an activation function for the output weights. The weight derived in this way was multiplied by the finite element analysis result to update the cable length in the simulation [26,27,28]. The error calculations obtained by comparing the updated finite element analysis results with the experimental results are shown in Figure 11. In Formula (4), h represents a weight derived through machine learning using the cylinder diameter, cable diameter and tension as input variables for updating the distance between cable markers. $\beta_{n}$ represents a node’s weight for each input variable. In Equation (5), $S_{FEM}$ is the distance between markers in the finite element analysis, and $S_{EXP}$ represents the distance between markers calculated through experiments.

(4) $$h=f(D_{cylinder},D_{cable},T)=f(x_{1},x_{2},x_{3})=\beta_{0}+\beta_{1}x_{1}+\beta_{2}x_{2}+\beta_{3}x_{3}$$

(5) $$ℒ=MSE(h\times S_{FEM}-S_{EXP})$$

As shown in Table 8, it was confirmed that the relative error of each cable decreased by up to 7% after updating the finite element analysis result. When the cable diameter was 2 mm, which was the smallest cable diameter and had very low axial rigidity, the error rate was larger compared with the other diameters. However, when the axial rigidity was relatively large, the error in the updated cable length was very small. In the first length, there was a large error in the process of fixing the cable to the cylinder, which hindered a smooth update. In order to increase accuracy, more data could be obtained by lengthening the cable length and increasing the experimental and finite element analysis time, leading to more accurate results.

## 5. Conclusions

The objective of this study was to develop a reliable method for estimating the physical properties of new materials by matching the behavior of a flexible cable wound around a cylinder through both finite element analysis and experiments. To achieve this, a finite element analysis model for the flexible cable winding operation was created, and the analysis was conducted based on the material properties and input/output conditions of the cable. To verify the accuracy of the finite element analysis results, experimental devices were designed and manufactured to behave in the same way. However, there were some differences between the experimental and analysis results, so an indicator had to be developed through trial and error to properly match the two cases. Many errors occurred during the experiment, depending on the analysis and experimental conditions. To improve this, weights that could update the cable analysis results were derived through optimization. Finally, deep learning was used to update the error caused by material properties by multiplying the finite element analysis result by the weight. In this way, even when the exact physical properties of the material were unknown, finite element analysis could be performed, thereby enhancing the performance of the analysis.

## 6. Future Work

The focus of this study was to enhance the performance of finite element analysis in cases where the physical properties of materials are unknown. The proposed method involves using deep learning techniques to update the errors between finite element analysis data and experimental data, which is achieved through a weighting strategy. This strategy enables the updating of analysis results, even when the data preprocessing, initial conditions, and boundary conditions are not optimized for simulation analysis. Nonlinear materials, such as flexible cables, often require time-consuming and expensive experiments, such as tensile tests, to determine the material properties needed for finite element analysis. This process is further complicated by the difficulty in accurately identifying properties due to the nonlinear nature of these materials. Moreover, modifying the analysis model for changes in cable type or diameter can be cumbersome. To improve the performance of finite element analysis, the final analysis result can be updated through multiplication by the weight. However, the error in the preprocessing stage cannot be completely eliminated from the perspective of finite element analysis. Therefore, a fundamental solution based on the material’s properties cannot be proposed, and further research is needed to automate the process of updating material properties by conducting experiments and finite element analysis on a wider range of cables and cases.

## Figures and Tables

**Table 1:** Conditions of finite element analysis and experiment for silicone rubber cable.

Case	Cylinder Diameter (mm)	Wire Diameter (mm)	Tension (MPa)
1	30	2	0.6245
2			1.249
3		4	0.1561
4			0.3122
5		6	0.06939
6			0.1388
7	60	2	0.6245
8			1.249
9		4	0.1561
10			0.3122
11		6	0.06939
12			0.1388

**Table 2:** Comparison of rotation angles and z-coordinates for markers in the simulations and experiments when the cable diameter was 2 mm.

Cable Diameter: 2 mm		Cylinder Diameter (mm)							
		30				60			
Tension (MPa)	Distance	Simulation		Experiment		Simulation		Experiment	
		$\theta_{n}(rad)$	$Z_{n}(mm)$	$\theta_{n}(rad)$	$Z_{n}(mm)$	$\theta_{n}(rad)$	$Z_{n}(mm)$	$\theta_{n}(rad)$	$Z_{n}(mm)$
0.6245	$P_{0}-P_{1}$	6.26	7.01	7.1	6.72	7.05	8.31	8.38	11.62
	$P_{0}-P_{2}$	12.02	17.69	14.66	17.51	13.91	22.07	18.1	34.62
	$P_{0}-P_{3}$	18.31	30.54	22.38	32.23	18.94	39.66	27.75	60.9
	$P_{0}-P_{4}$	24.64	45.26	30.32	50.15	25.75	57.86	-	-
1.249	$P_{0}-P_{1}$	6.85	7.24	7.1	6.91	7.06	8.38	8.45	11.84
	$P_{0}-P_{2}$	13.09	17.33	15.03	19.5	13.34	22.23	19.15	37.55
	$P_{0}-P_{3}$	19.38	29.73	23.74	38.11	19.56	39.75	30.58	67.28
	$P_{0}-P_{4}$	25.65	44.8	-	-	25.78	58.07	-	-

**Table 3:** Comparison of rotation angles and z-coordinates for markers in the simulations and experiments when the cable diameter was 4 mm.

Cable Diameter: 4 mm		Cylinder Diameter (mm)							
		30				60			
Tension (MPa)	Distance	Simulation		Experiment		Simulation		Experiment	
		$\theta_{n}(rad)$	$Z_{n}(mm)$	$\theta_{n}(rad)$	$Z_{n}(mm)$	$\theta_{n}(rad)$	$Z_{n}(mm)$	$\theta_{n}(rad)$	$Z_{n}(mm)$
0.6245	$P_{0}-P_{1}$	8.48	5.68	6.23	3.67	6.85	9.53	6.6	9.13
	$P_{0}-P_{2}$	12.73	16.33	12.34	14.66	13.11	23.32	13.37	23.13
	$P_{0}-P_{3}$	19.07	28.31	18.38	28.68	19.32	40.2	20	41.23
	$P_{0}-P_{4}$	25.34	42.29	24.38	45.01	25.55	58.21	26.7	59.95
1.249	$P_{0}-P_{1}$	6.31	5.66	6.28	4.5	6.86	9.69	6.77	8.21
	$P_{0}-P_{2}$	12.49	16.33	12.52	14.86	13.13	23.46	13.56	22.14
	$P_{0}-P_{3}$	18.79	28.28	18.78	28.39	19.31	40.27	20.32	39.73
	$P_{0}-P_{4}$	25.06	42.19	24.96	43.6	25.55	58.35	27.17	59.12

**Table 4:** Comparison of rotation angles and z-coordinates for markers in the simulations and experiments when the cable diameter was 6 mm.

Cable Diameter: 6 mm		Cylinder Diameter (mm)							
		30				60			
Tension (MPa)	Distance	Simulation		Experiment		Simulation		Experiment	
		$\theta_{n}(rad)$	$Z_{n}(mm)$	$\theta_{n}(rad)$	$Z_{n}(mm)$	$\theta_{n}(rad)$	$Z_{n}(mm)$	$\theta_{n}(rad)$	$Z_{n}(mm)$
0.6245	$P_{0}-P_{1}$	6.31	6.35	5.76	5.85	9.54	10.16	5.99	8.87
	$P_{0}-P_{2}$	12.49	16.55	11.47	12.73	12.23	22.62	12.13	19.95
	$P_{0}-P_{3}$	18.79	28.25	17.05	24.88	18.55	39.1	18.29	34.5
	$P_{0}-P_{4}$	25.05	41.68	22.74	42.3	24.9	56.67	24.43	49.1
1.249	$P_{0}-P_{1}$	6.66	6.37	5.88	5.19	6.66	10.33	6.06	9.77
	$P_{0}-P_{2}$	12.9	16.54	11.64	11.5	12.9	22.82	12.23	21.83
	$P_{0}-P_{3}$	19.15	28.22	17.28	23.93	19.15	39.16	18.5	36.56
	$P_{0}-P_{4}$	25.37	41.72	23.11	38.11	25.37	56.75	24.73	51.66

**Table 5:** Calculations of the cable length to each marker for a cylinder diameter of 30 mm.

Case		Cable Length (mm)			
		$S_{1}$	$S_{2}$	$S_{3}$	$S_{4}$
Original length		100	200	300	400
1	Simulation	100.37	193.14	294.51	396.77
	Experiment	113.8	235.21	359.53	487.71
2	Simulation	109.87	210.16	311.51	412.76
	Experiment	113.81	241.27	381.75	-
3	Simulation	144.23	217.03	325.37	432.92
	Experiment	105.97	210.29	313.77	416.9
4	Simulation	107.42	212.92	320.7	428.04
	Experiment	106.85	213.36	320.52	426.55
5	Simulation	113.75	225.37	339.38	452.89
	Experiment	103.84	206.85	307.91	411.5
6	Simulation	120.1	232.8	345.78	458.55
	Experiment	105.97	209.84	311.96	417.72

**Table 6:** Calculations of the cable length to each marker for a cylinder diameter of 60 mm.

Case		Cable Length (mm)			
		$S_{1}$	$S_{2}$	$S_{3}$	$S_{4}$
Original length		200	400	600	800
7	Simulation	218.68	431.86	588.49	800.24
	Experiment	260.04	562.17	862.4	-
8	Simulation	219.06	414.05	607.8	801.32
	Experiment	262.22	594.84	950.36	-
9	Simulation	219.49	420.03	619.47	819.54
	Experiment	211.4	428.46	641.33	856.5
10	Simulation	219.76	420.72	619.39	819.62
	Experiment	216.8	434.48	651.45	871.45
11	Simulation	314.92	404.32	613.55	823.61
	Experiment	197.87	400.79	604.56	807.68
12	Simulation	220.11	426.33	633.03	839.1
	Experiment	200.22	404.18	611.59	817.72

**Table 7:** Percentage error for cable length in each case.

Case	Percentage Error of Cable length (%)			
	$S_{1}$	$S_{2}$	$S_{3}$	$S_{4}$
1	13.38	21.78	22.08	22.92
2	3.59	14.8	22.55	-
3	26.53	3.11	3.57	3.7
4	0.53	0.21	0.06	0.35
5	8.71	8.22	9.27	9.14
6	11.77	9.86	9.78	8.9
7	18.91	30.17	46.54	-
8	19.7	43.66	56.36	-
9	3.69	2.01	3.53	4.51
10	1.35	3.27	5.18	6.32
11	37.17	0.87	1.47	1.93
12	9.04	5.2	3.39	2.55

**Table 8:** Percentage error of cable length for updated finite element analysis.

Case	Updated Percentage Error of Cable length (%)			
	$S_{1}$	$S_{2}$	$S_{3}$	$S_{4}$
1	2.56	4.66	4.91	5.64
2	16.05	6.96	0.68	-
3	24.28	0.14	0.61	0.75
4	0.28	1.02	0.76	0.47
5	1.04	1.59	0.42	0.57
6	3.1	1.01	0.92	0.04
7	14.23	6.11	5.7	-
8	19.92	3.89	4.6	-
9	8.76	3.37	1.92	0.99
10	8.26	3.97	2.2	1.13
11	34.09	3.98	3.36	2.87
12	5.45	1.46	0.42	1.29

## Data Availability

Not applicable.
